# Comparison of clinicopathological features and survival in triple-negative and non-triple-negative breast cancer patients in Tanzania

**DOI:** 10.1093/oncolo/oyaf345

**Published:** 2025-11-18

**Authors:** Eulade Rugengamanzi, Nazima Dharsee, Emmanuel L Lugina, Jesse Jonathan Kashabano, Gad Murenzi, Alan Paciorek, Godfrey Malangwa, Mathias Banzi, Glory Makupa, Godwin Nnko, Leila Mwakipunda, Amie Y Lee

**Affiliations:** Academic, Research, and Consultancy Unit, Ocean Road Cancer Institute, Dar es Salaam, Tanzania; Department of Clinical Oncology, Muhimbili University of Health and Allied Sciences, Dar es Salaam, Tanzania; Department of Oncology, The Butaro Cancer Center of Excellence, Butaro, Rwanda; Clinical Division, Medical School, University of Global Health Equity (UGHE), Butaro, Rwanda; Einstein-Rwanda Research and Capacity Building Program, Research for Development (RD Rwanda), Kigali, Rwanda; Academic, Research, and Consultancy Unit, Ocean Road Cancer Institute, Dar es Salaam, Tanzania; Department of Clinical Oncology, Muhimbili University of Health and Allied Sciences, Dar es Salaam, Tanzania; Academic, Research, and Consultancy Unit, Ocean Road Cancer Institute, Dar es Salaam, Tanzania; Department of Clinical Oncology, Muhimbili University of Health and Allied Sciences, Dar es Salaam, Tanzania; Oncology departement, Benjamin Mkapa Hospital, Dodoma, Tanzania; Einstein-Rwanda Research and Capacity Building Program, Research for Development (RD Rwanda), Kigali, Rwanda; Department of Pathology, Muhimbili University of Health and Allied Sciences, Dar es Salaam, Tanzania; Department of Epidemiology and Biostatistics, UCSF, San Francisco, CA, United States; Internal Medicine departement , Muhimbili National Hospital-Mlonganzila, Dar es Salaam, Tanzania; Department of Oncology, Mbeya Zonal Referral Hospital, Mbeya, Tanzania; Academic, Research, and Consultancy Unit, Ocean Road Cancer Institute, Dar es Salaam, Tanzania; Department of Clinical Oncology, Muhimbili University of Health and Allied Sciences, Dar es Salaam, Tanzania; Department of Oncology, Kilimanjaro Christian Medical Center (KCMC), Moshi, Tanzania; Academic, Research, and Consultancy Unit, Ocean Road Cancer Institute, Dar es Salaam, Tanzania; Department of Clinical Oncology, Muhimbili University of Health and Allied Sciences, Dar es Salaam, Tanzania; Department of Oncology, Kilimanjaro Christian Medical Center (KCMC), Moshi, Tanzania; Department of Oncology, Kilimanjaro Christian Medical Center (KCMC), Moshi, Tanzania; Department of Radiology and Biomedical Imaging, University of California, San Francisco (UCSF) San Francisco, United States

**Keywords:** triple-negative breast cancer (TNBC), overall survival, sub-Saharan Africa, breast cancer subtypes

## Abstract

**Purpose:**

Breast cancer is a heterogeneous disease with a wide spectrum of subtypes, each with distinct biological features. Data on tumor subtypes in East Africa are sparse despite the rising incidence of breast cancer in this region. We aimed to determine the prevalence of triple-negative breast cancer (TNBC) and to compare clinicopathological features, overall survival (OS), and factors affecting OS of patients with TNBC and non-TNBC in Tanzania.

**Methods:**

This retrospective nested case–control study included patients with histologically proven breast cancer treated at Ocean Road Cancer Institute (ORCI) in Tanzania from January 2018 to December 2019. Logistic regression was used to determine factors associated with TNBC, and Cox proportional hazards regression was used to determine the factors independently associated with overall survival.

**Results:**

TNBC constituted 23.3% of all breast cancer diagnoses. Patients in the TNBC group were younger (median age 46 vs 53 years, *P* < .001) and more often presented with stage IV disease (12% vs 4%, *P* = .001). Three-year OS was significantly lower in the TNBC group (36%) compared to the non-TNBC group (57%) (*P* = .004) and remained lower for the TNBC group across every tumor stage. Brain metastasis was higher in the TNBC group (34% vs 7%, *P* < .001). Factors associated with improved survival included shorter symptom duration (0.53 [0.36-0.78]), earlier stage (0.23 [0.12-0.46]), use of neoadjuvant chemotherapy (0.59 [0.40-0.86]), and surgery with clean margins (0.34 [0.19-0.60]).

**Conclusions:**

TNBC accounted for nearly a quarter of the breast cancers at ORCI. TNBC was associated with younger age, more aggressive clinicopathologic features, and worse survival. Efforts toward earlier diagnosis and optimized therapies will be critical to improving TNBC outcomes in Tanzania.

Implications for Practice This study highlights the urgent need for earlier detection and tailored treatment strategies for triple-negative breast cancer (TNBC) in Tanzania. The high prevalence of TNBC, its association with younger age and advanced disease at presentation, and poorer survival outcomes underscore the importance of strengthening breast cancer screening programs and public awareness campaigns. Clinicians should prioritize timely diagnosis and consider neoadjuvant chemotherapy and surgery with clean margins as critical interventions to improve survival. Additionally, the findings support the development of multidisciplinary care models and investment in pathology infrastructure to ensure accurate tumor subtyping and guide treatment decisions. These efforts are essential to address disparities in breast cancer outcomes and improve care for women across sub-Saharan Africa.

## Introduction

Breast cancer is the leading cause of cancer-related deaths among women worldwide and its incidence is rising, including in sub-Saharan Africa.^1^^,^^2^ According to 2020 global cancer data, nearly 2.5 million cases of newly diagnosed breast cancer occurred globally, with 700,000 total deaths.^3^

Breast cancer is a heterogeneous disease with a wide spectrum of subtypes. Based on gene expression profiling, breast cancer can be categorized into 5 molecular subtypes: luminal A, luminal B, normal breast-like, those characterized by human epidermal growth factor receptor 2 (HER2/neu) overexpression, and basal-like.^4^^,^^5^ Each subtype is associated with distinct biological features, prognostic indicators, and differences in response to treatment modalities.^6^ Most triple-negative breast cancers (TNBCs) are basal-like.^7^ According to the American Society of Clinical Oncology guideline and the latest St Gallen consensus, TNBC is defined as a breast tumor without expression of estrogen (ER), progesterone receptor (PR), and neither expression nor amplification of HER2/neu.^8^^,^^9^

TNBC represents a distinctive and unique subtype within the spectrum of breast cancer characterized by more aggressive behavior, higher recurrence, and poorer survival compared to non-triple-negative breast cancer.^10^ The poorer prognosis of TNBC is in part due to lack of targeted therapy compared to other molecular subtypes managed with hormonal therapy like tamoxifen and aromatase inhibitors that treat hormone receptor-positive cancers and targeted therapies like trastuzumab and pertuzumab that treat HER2/neu + cancers.^11^ Given the absence of well-defined therapeutic targets, the prevailing standard of care for TNBC involves a regimen combining anthracycline and taxane-based chemotherapy, along with surgical intervention, with or without radiotherapy.^12^

A study conducted in the United States found that TNBC constituted 20% of breast cancers diagnosed in Black Americans.^12^ In contrast, TNBC has been shown to constitute only 8% of breast cancers among Japanese women and 12% of breast cancers in Sweden. While research into TNBC in sub-Saharan Africa is nascent, higher rates have been reported in Ghana and Nigeria, ranging from 22% to 58% of breast cancer cases.^13^^,^^14^ TNBCs are also more commonly diagnosed in younger patients compared to non-TNBC, as demonstrated across various studies.^15^

In Tanzania and East Africa in general, very limited information has been reported on clinicopathological differences and outcomes by tumor molecular subtypes in this region. Therefore, in this study, we aimed to investigate the prevalence of TNBC and compare clinicopathological features, overall survival (OS), and factors affecting OS for TNBC vs non-TNBC in Tanzania.

## Methodology

### Study design and participants

We conducted a retrospective nested case–control study of patients with histologically confirmed breast cancer who received care at Ocean Road Cancer Institute (ORCI) in Dar es Salaam, Tanzania, between January 2018 and December 2019. Patients were identified through a review of the hospital registry. In the cohort, we included participants who were ≥18 years of age at the time of diagnosis and had an immunohistochemistry (IHC) report available. Patients who were not permanent residents of Tanzania and patients who did not receive clinical care at ORCI were excluded. In the case–control sample selected among the cohort, we included all patients determined to be TNBC as the cases and a random set of non-TNBC patients as the controls.

All study procedures were approved by the ORCI research ethics committee and by institutional review board of the Muhimbili University of Health and Allied Sciences (MUHAS-REC-09-2022-1375).

### Study setting

ORCI is Tanzania’s largest national cancer referral hospital. It offers comprehensive cancer treatment, including chemotherapy and radiotherapy. For patients who undergo neoadjuvant chemotherapy, the standard regimen used during our study period consisted of an AC combination (doxorubicin/cyclophosphamide) as first-line chemotherapy, followed by 4 rounds of docetaxel or paclitaxel.

Pathologic diagnoses were performed at the Central Pathology Laboratory at Muhimbili National Hospital (MNH). IHC for breast cancer specimens was done by cutting 3 μm tissue sections, staining with DAKO antibodies for ER, PR, and human epidermal growth factor receptor 2 (HER2), and employing a scoring system for positivity. HER2 status was determined based on membrane reactivity and cell percentage. For HER2-equivocal (HER2 2+) cases, we confirmed HER2 status using fluorescence in situ hybridization (FISH), classifying FISH-positive cases as HER2-positive and FISH-negative cases as HER2-negative, and 2 pathologists always confirm the results.

### Data collection

Demographic and clinicopathologic data were abstracted from the hospital-based electronic record system “Inaya” at ORCI. Demographic variables collected included age, gender, family history of cancer, menopausal status, marital status, and insurance status. Clinical variables collected included body mass index, symptom duration, disease stage at presentation, histology, pathological type, tumor grade, tumor size, lymph node involvement, lymphovascular invasion, and treatment details. Vital status data were obtained from the electronic medical record when available. In cases in which the vital status was unavailable in the electronic medical record, attempts were made to contact the patient or their caregivers via telephone to inquire about the patient’s status.

All patients with a breast cancer diagnosis treated at ORCI were first identified. Then, using a nested case–control study design, all cases of TNBC and a random selection of non-TNBC according to IHC results were selected for further analyses. We required one control non-TNBC patient for every identified case TNBC patient to ensure data collection was feasible with limited resources. We designated a patient as non-TNBC if they are either Hormone positive or Her2 positive. This study design was chosen to efficiently estimate prevalence of TNBC using the cohort and also test for factors associated with TNBC status and overall survival using the case–control subset. We believe this design entailed minimal bias because both cases and controls were random and are representative of the target population of breast cancer patients across Tanzania.

### Statistical analysis

The frequency of TNBC was determined by taking the ratio of the number of TNBC cases over the total number of breast cancer cases treated at ORCI between 2018 and 2019. Patient and disease characteristics were compared across the 2 hormone status groups, TNBC and non-TNBC. The Chi-square test was utilized to compare percentages. Means were compared using the Kruskal–Wallis test. OS was defined as the time from the date of the medical diagnosis of the disease by pathological confirmation to the date of the death or last follow-up. The Kaplan–Meier technique was used to create survival curves starting at the initial diagnosis. For the comparison of OS across the 2 groups, we used the log-rank test.

To determine what factors independently affect survival, we used Cox regression models. Our primary goal is to measure the magnitude of the effect of TNBC hormone status on overall survival. We measured the effects of other factors that we expected, based on previous studies, would be associated with survival, then added a product term with TNBC hormone status to determine if the effect is different for patients with and without TNBC. We chose the final multivariable model using Akaike’s information criterion and forced the model to include age because it is proven to be associated with survival and to include TNBC hormone status because that was the primary aim of this study. All analyses were performed using Stata 17 (StataCorp. 2023). A *P*-value of <.05 was considered statistically significant.

## Results

A total of 761 cases of breast cancer were identified at ORCI during the study period. Of these cases, 720 had an IHC report available in their medical charts for review. Among the 720 patients eligible for inclusion in this analysis, 168 were reported as TNBC, with a prevalence of 23% (95% CI 20-27). From the 720 cases, we performed a random 1:1 selection of 124 cases of TNBC and 124 cases of non-TNBC for our final analysis. The non-TNBC group included hormone receptor-positive/HER2-negative cases (59/124, 47.6%), hormone receptor-positive/HER2-positive cases (48/124, 38.7%), and hormone receptor-negative/HER2-positive cases (17/124, 13.7%).

### Sociodemographic features

The mean age at diagnosis in the TNBC group was 46 years (range 19-82) compared to 53 years (range 29-87) in the non-TNBC group (*P* < .001). The TNBC group had a greater proportion of patients younger than 35 years old (22% vs 3%, *P* < 0.001) and a greater proportion of premenopausal women (48% vs 31%, *P* = .006) compared to the non-TNBC group. The TNBC group had a higher proportion of patients who reported a family history of breast cancer (38% vs 4%, *P* < .001) among the nearly half of patients analyzed who had known family history status. A summary of all data comparing the social and demographic characteristics of the 2 groups is presented in **Table 1**.

**Table 1.** Sociodemographic characteristics by hormone status as triple-negative breast cancer (TNBC) or non-triple-negative breast cancer.

Characteristic *N* (%)	Value	Overall	Non-TNBC	TNBC	*P*-value^a^
Total		248	124	124	
Age at diagnosis	Median [range]	50 [19-87]	53 [29-87]	46 [19-82]	<.001
Age group	<35	31 (13)	4 (3)	27 (22)	<.001
	35-60	159 (64)	83 (67)	76 (61)	
	>60	58 (23)	37 (30)	21 (17)	
Menopausal status	Pre	99 (40)	39 (31)	60 (48)	.006
	Post	149 (60)	85 (69)	64 (52)	
Marital status	Married	181 (73)	91 (73)	90 (73)	.918
	Single	17 (7)	8 (6)	9 (7)	
	Divorced	12 (5)	7 (6)	5 (4)	
	Widowed	38 (15)	18 (15)	20 (16)	
Health insurance	Yes	91 (37)	48 (39)	43 (35)	.510
	No	157 (63)	76 (61)	81 (65)	
Symptom duration	≤6	111 (45)	63 (51)	48 (39)	.055
(months)	7 or more	137 (55)	61 (49)	76 (61)	
BMI (kg/m^2^)	<18	6 (2)	5 (4)	1 (<1)	.175
	18-25	167 (67)	79 (64)	88 (71)	
	>25	75 (30)	40 (32)	35 (28)	
Family history of	Yes	18 (15)	3 (4)	15 (38)	<.001
	No	105 (85)	81 (96)	24 (62)	
breast cancer	Unknown	125	40	85	

a Chi-square test or Kruskal–Wallis test; *P*-values are based on known values.

### Clinicopathological features

A large proportion of patients were diagnosed at an advanced stage (AJCC stage III or IV) in both the TNBC group (77%) and the non-TNBC group (68%), and the difference was not statistically significant (*P* = .088). A greater proportion of TNBC patients had stage IV disease at diagnosis compared to non-TNBC (12% vs 4%, *P* = .020), and a smaller proportion of TNBC had stage II compared to non-TNBC (15% vs 31%, *P* < .001). More TNBC patients had higher histologic grade (grade 3) compared to non-TNBC (50% vs 30%, *P* = .002). Among TNBC patients, 75% had lymphovascular invasion reported vs 54% of non-TNBC patients (*P* = .001). A total of 215 out of the 248 patients underwent surgery as part of their treatment, and 211 had available data on the status of surgical margins. More TNBC patients’ surgeries resulted in positive margins compared to non-TNBC patients (51% vs 28%, *P* = .001) (Table 2).

**Table 2.** Clinicopathological features and treatments by hormone status as triple-negative breast cancer (TNBC) or non-triple-negative breast cancer.

Characteristic *N* (%)	Value	Overall	Non-TNBC	TNBC	*P*-value^a^
Total		248	124	124	
AJCC staging	I	11 (4)	2 (2)	9 (7)	.001
	II	57 (23)	38 (31)	19 (15)	
	III	160 (65)	79 (64)	81 (65)	
	IV	20 (8)	5 (4)	15 (12)	
Pathological type	Ductal	213 (86)	106 (85)	107 (86)	.873
	Lobular	20 (8)	11 (9)	9 (7)	
	Others	15 (6)	7 (6)	8 (6)	
Axillary lymph nodes	Positive	166 (67)	80 (65)	86 (69)	.418
	Negative	82 (33)	44 (35)	38 (31)	
Tumor size (mm)	Median [Range]	5.2 [0-5.8]	5.2 [0-5.8]	5.4 [0-5.8]	.134
Histological grade	I-II	143 (60)	81 (70)	62 (50)	.002
	III	96 (40)	35 (30)	61 (50)	
	Unknown	9	8	1	
Margins	Clean	128 (61)	78 (72)	50 (49)	.001
	Positive	83 (39)	31 (28)	52 (51)	
	Unknown	4	3	1	
Lymphovascular	Yes	141 (64)	60 (54)	81 (75)	.001
Invasion	No	78 (36)	51 (46)	27 (25)	
	Unknown	29	13	16	
Neoadjuvant chemotherapy	Yes	166 (67)	99 (80)	67 (54)	<.001
	No	82 (33)	25 (20)	57 (46)	
Surgery	Yes	215 (87)	112 (90)	103 (83)	.092
	No	33 (13)	12 (10)	21 (17)	
Radiation	Yes	167 (67)	84 (68)	83 (67)	.892
	No	81 (33)	40 (32)	41 (33)	

a Chi-square test or Kruskal–Wallis test; *P*-values are based on known values.

**Figure 1.**
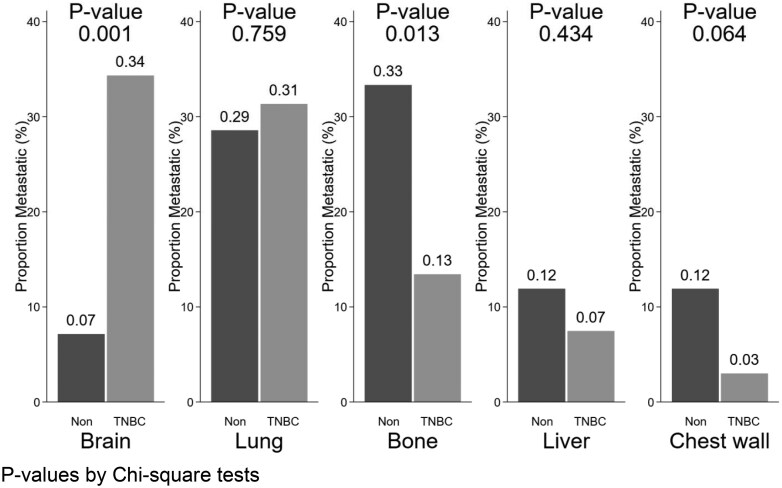
Metastasis location by hormone status as triple-negative breast cancer (TNBC) or non-triple-negative breast cancer. *P*-values by chi-square tests.

Among 228 patients without distant metastasis at initial presentation (109 for TNBC and 119 for non-TNBC), 109 (48%) developed metastasis after primary cancer treatment. In the TNBC group, the rate of developing distant metastasis after initial cancer diagnosis was significantly higher than in the non-TNBC group (61% vs 35%, *P* < .001). Among the 67 patients with TNBC who developed distant metastases, the most common initial site of metastasis was brain (34%), followed by lung (31%). Among the 42 patients with non-TNBC who developed distant metastases, the most common site of metastasis was in bone (33%), followed by lung (29%). Development of brain metastasis was higher in the TNBC group compared to non-TNBC (34% vs 7%, *P* < .001). Sites of distant metastases are summarized in Figure 1.

### Survival data

The median follow-up time was 33 months (range: 1-64 months), during which 145 deaths were observed (86 in the TNBC group and 62 in the non-TNBC group). The median OS among patients with TNBC was 25 months (95% CI 22-33 months). The median OS among patients with non-TNBC was 47 months (95% CI 32-52 months).

**Figure 2.**
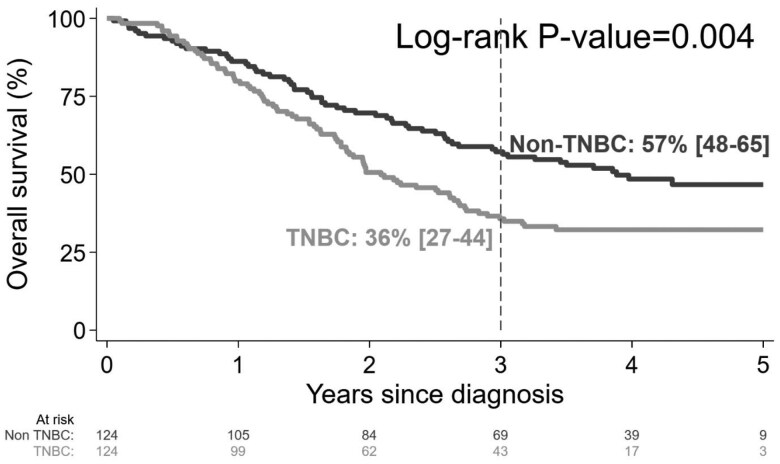
Overall survival by hormone status as triple-negative breast cancer (TNBC) or non-triple-negative breast cancer.

The 3-year OS was significantly lower in the TNBC group (36%) compared to the non-TNBC group (57%) (*P* = .004), as shown in Figure 2. The 3-year OS for patients with TNBC was lower than for patients with non-TNBC across every tumor stage; survival rates were 88% vs 100% for stage 1, 83% vs 92% for stage 2, 24% vs 40% for stage 3, and 6% vs 20% for stage 4, respectively (*P* < .001).

To measure the independent effect of TNBC status on OS, a multivariable Cox regression model was fit with TNBC status and other predictors of OS including age, stage, symptom duration, use of neoadjuvant chemotherapy, and surgery with margins status. All other potential predictors, as shown in Table 3, were not independently associated with OS. The effects of every exposure were uniform between TNBC and non-TNBC patients (all interaction term *P*-values >.05, data not shown). After adjustment for other predictors, such use of neoadjuvant chemotherapy and surgery, there was no statistically significant differences in OS between TNBC and non-TNBC (adjusted HR: 1.00 [95% CI: 0.67-1.50, *P* = .988]). There was evidence in our sample that other factors were associated with survival. Risk of mortality was reduced 47% for patients with symptom duration shorter than 7 months (HR 0.53 95% CI [36-78]), 77% for patients with stage 1-2 disease (HR 0.23 95% CI [0.12-0.46]), 41% for patients who received neoadjuvant chemotherapy (HR 0.59 95% CI [0.40-0.86]), 66% for patients who underwent surgery with negative margins (HR 0.34 95% CI [0.19-0.60]).

**Table 3.** Factors associated with overall survival.

Characteristic	Value	Univariate analysis			Multivariate analysis		
		HR	95% CI	*P*-value	HR	95% CI	*P*-value
Hormone status	TNBC vs Non-TNBC	1.63	1.17-2.27	.004	1.00	0.67-1.50	.988
Age at diagnosis	Additional 5 years	0.97	0.91-1.04	.432	1.01	0.94-1.08	.845
Health Insurance	Yes vs No	0.41	0.28-0.60	<.001	NA		
Symptom duration	≥7 vs <7 months	3.13	2.17-4.53	<.001	1.90	1.29-2.80	.001
Histology	Lobular vs Ductal	0.98	0.54-1.77	.943	NA		
	Other vs Ductal	1.02	0.52-2.02	.944	NA		
Histological grade	III vs I-II	4.66	3.29-6.60	<.001	NA		
LVI	Yes vs No	4.06	2.64-6.26	<.001	NA		
Advanced stage at diagnosis	III-IV vs I-II	8.97	4.71-17.11	<.001	4.29	2.10-8.45	<.001
Neoadjuvant chemotherapy	Yes vs No	0.32	0.23-0.44	<.001	0.59	0.40-0.86	.005
Surgery and margins	Yes and clean margins vs No surgery	0.09	0.05-0.14	<.001	0.34	0.19-0.60	<.001
	Yes and positive margins vs No surgery	0.49	0.32-0.75	.001	0.95	0.58-1.57	.844
Radiation and type	Conv vs No radiation	0.24	0.17-0.36	<.001	0.37	0.24-0.57	<.001
	Hypo vs No radiation	0.18	0.11-0.28	<.001	0.35	0.21-0.59	<.001

Abbreviations: CI, confidence interval; HR, hazard ratio; LVI, lymphovascular invasion; trt, treatment; hypo, hypofractionation; conv, convention.

## Discussion

To the best of our knowledge, our study represents the largest series of patients with TNBC to date in the Tanzanian context. TNBC accounted for nearly a quarter of all breast cancer diagnoses at ORCI between 2018 and 2019. TNBC was associated with significantly younger age, more advanced stage at presentation, more aggressive pathologic features, and poorer survival outcomes compared to non-TNBC patients. The poorer survival for TNBC patients seen on univariate analysis was no longer evident when either a measure of surgery with clean margins or neoadjuvant chemotherapy was added to the model, suggesting that survival can be improved with appropriate treatments.

The high prevalence of TNBC in this study is in accordance with prior literature from sub-Saharan Africa.^16^ The prevalence of TNBC in Africa is ranged from 20% up to 50% of breast cancer cases.^13^^,^^17^^,^^18^ This highlights the known variations in TNBC occurrence among different racial populations. The higher prevalence of TNBC among Africans compared to Caucasians or Asians suggests that geographical and/or racial variations in tumor biology may exist. The etiology of this variability is unknown but may be due to genetic variations and/or environmental factors.^19^^,^^20^

Tanzanian patients in the TNBC group were younger than the non-TNBC group. Our results are in keeping with prior studies showing that the age at diagnosis of TNBC is lower than that of non-TNBC.^21^^,^^22^ In addition, studies have shown TNBC occurs at younger ages in the populations of African ancestry compared to Caucasians; this may be partly explained by different tumor biology, such as associated genetic mutations and gene expression patterns in individuals of African ancestry.^23^^,^^24^ The younger age of diagnosis of this aggressive disease in Tanzania highlights the need for interventions for much earlier detection and treatment.

We found that more patients with TNBC had a family history of breast cancer compared to non-TNBC. Prior studies showed similar results. A study by Rweyamamu et al. in Tanzania and Qui et al. in China found that 24% and 11% of patients with TNBC had a history of breast cancer in the family, which was significantly higher than non-TNBC patients.^19^^,^^21^ Although we did not assess gene mutation status, as genetic testing is not widely available in Tanzania, this association of TNBC and family history may partly be due to inherited cancer susceptibility genetic mutations. Studies have shown a strong association between TNBC and BRCA mutations^25^; in China, BRCA mutations accounted for 8% of breast cancer diagnoses, and the majority were associated with TNBC.^26^ Similarly, a study in Nigeria found that BRCA mutations were more common with TNBC compared with non-TNBC tumors (87.7% vs 21.6, *P* < .001).^27^ This highlights the need for subsequent investigations into the prevalence of BRCA mutations in Tanzania.

In our study, we observed no statistically significant difference in BMI distribution between TNBC and non-TNBC groups (*P* = .175). The majority of patients fell within the normal BMI range (18-25 kg/m^2^), with a slightly higher proportion in the TNBC group (71%) compared to non-TNBC (64%). Obesity (>25 kg/m^2^) was present in 32% of non-TNBC and 28% of TNBC cases, while underweight individuals (<18 kg/m^2^) were rare in both groups. Although obesity is a well-established risk factor for hormone receptor-positive breast cancer, its role in TNBC remains uncertain. Some studies have suggested that visceral fat may contribute to TNBC risk, particularly in African American women, possibly through inflammatory pathways and insulin resistance.^28^^,^^29^ However, other analyses, including our findings, indicate that BMI alone may not be a key distinguishing factor for TNBC risk, especially in African populations where genetic predisposition and other lifestyle factors may play a more significant role.^30^ This highlights the need for more studies to better understand risk factors for TNBC in African women.

Our study also found that TNBC is associated with a more advanced clinical stage at presentation, compared to non-TBNC. This is similar to prior studies. For example, Lin et al. found the majority of patients with TNBC present with advanced stage compared to non-TNBC.^31^ There was also a higher rate of brain metastasis in the TNBC group, in keeping with the known metastatic tendencies, or organotropism, of TNBC.^32^^,^^33^

Survival outcomes differed significantly between the groups; 3-year OS was lower in the TNBC group compared to the non-TNBC group. This difference is similar to other studies.^19^^,^^34^ A study of 1944 breast cancer patients in the United Kingdom found that the DFS and OS rates for TNBC were significantly poorer than those for non-TNBC. Studies in the United States^35^^,^^36^ found the 5-year DFS rate in the TNBC was 67% compared to 82% in the non-TNBC. Another study in China^37^ revealed that 5-year DFS and 5-year OS rates in the TNBC were 73.7% and 88.5%, respectively, as opposed to 80.8% and 92.8% in the non-TNBC, which all differed significantly. Furthermore, survival outcomes for TNBC are reported to be far poorer in low and middle-income counties (LMICs) compared to high-income countries; in Malawi, 1-year OS is only 57%,^38^ in Mozambique, 3-year OS was 32%.^39^^,^^40^ These lower survival rates in LMICs could be attributed to patient characteristics, access to medical interventions, and the standard of care provided by hospitals. Increased efforts toward earlier diagnosis and optimized therapies are needed to improve the outcomes for patients with TNBC in sub-Saharan Africa.

Not surprisingly, we found that treatment with neoadjuvant chemotherapy and surgery were both associated with reducing mortality. This is in keeping with several studies and underscores the critical role of a multidisciplinary team and collaborative efforts for improved outcomes.^39^^,^^41^^,^^42^ Multiple studies from around the globe have previously shown that neoadjuvant therapy consistently increases OS for patients with TNBC.^39^^,^^41^

Our study has limitations. First, this was single-institution study and our findings may not be generalizable. That said, ORCI is Tanzania’s largest national cancer referral hospital and treats patients from all regions of the country. Second, this study was retrospective in nature. For example, we collected IHC reports retrospectively. Because we do not know the mean time of fixation time, this may have led to false negatives as studies have shown that a delay in fixation of more than 24 hours can affect the accuracy of ER IHC.^43^ Also, our study lacked data on complete pathological response (pCR), as this was not routinely reported during the study period. pCR has only very become a standard practice at our institution in 2024, and therefore, its inclusion was not feasible for the cohort analyzed.

## Conclusion

TNBC constitutes a large proportion of breast cancer cases at the Ocean Road Cancer Institute in Tanzania. This underscores the substantial burden of this aggressive tumor subtype in the region. TNBC patients were diagnosed at younger ages, with more advanced stages and higher grades. Furthermore, the 3-year OS in TNBC patients was significantly lower compared to non-TNBC patients. Longer symptom duration before diagnosis and treatment, more advanced stage, lack of neoadjuvant chemotherapy, and lack of surgery were associated with worse survival.

This study enhances the limited data on the clinical profile and survival disparities between TNBC and non-TNBC in the African context. To improve breast cancer outcomes, there is a critical need to enhance public awareness and improve healthcare access to facilitate early detection and treatment. Early diagnosis and intervention have the potential to significantly impact survival rates, especially for aggressive subtypes like TNBC. This will require a pragmatic framework to establish an effective multidisciplinary breast cancer care program, emphasizing collaboration, education, thorough auditing, adoption of best practices, research, and the organization of healthcare services.

## Data Availability

The data supporting the findings of this study are available from the corresponding author upon reasonable request. Due to ethical and privacy considerations, individual patient data are not publicly shared. Requests for access to de-identified data should be directed to Dr Eulade Rugengamanzi at veulade@gmail.com.
